# Pharmacopuncture in Korea: A Systematic Review and Meta-Analysis of Randomized Controlled Trials

**DOI:** 10.1155/2016/4683121

**Published:** 2016-01-06

**Authors:** Jimin Park, Hyangsook Lee, Byung-Cheul Shin, Myeong Soo Lee, Boryang Kim, Jong-In Kim

**Affiliations:** ^1^Department of Acupuncture and Moxibustion, College of Korean Medicine, Kyung Hee University, Seoul 02447, Republic of Korea; ^2^Acupuncture and Meridian Science Research Center, College of Korean Medicine, Kyung Hee University, Seoul 02447, Republic of Korea; ^3^Department of Rehabilitation Medicine, School of Korean Medicine, Pusan National University, Yangsan, Republic of Korea; ^4^Clinical Research Division, Korea Institute of Oriental Medicine, Daejeon, Republic of Korea

## Abstract

*Background*. Pharmacopuncture is a new form of acupuncture combining acupuncture with herbal medicine, and it has been used under various conditions in Korea. The aim of this study is to establish clinical evidence for the safety and efficacy of pharmacopuncture in Korea.* Methods*. We searched 9 databases and two relevant journals up to December 2014 using keywords, such as pharmacopuncture. All randomized, controlled trials evaluating pharmacopuncture under any conditions in Korea were considered.* Results*. Twenty-nine studies involving 1,211 participants were included. A meta-analysis of two studies on obesity showed that 5 to 8 weeks of pharmacopuncture reduced weight, waist circumference, and body mass index (BMI) more than normal saline injections. In the 5 studies of musculoskeletal conditions, 7 to 30 days of pharmacopuncture had additional effects on the reduction of pain intensity, and this benefit was maintained by limiting analyses to studies with a low risk of bias for randomization and/or allocation concealment.* Conclusions*. This systematic review suggests the potential of pharmacopuncture for obesity and musculoskeletal diseases. However, it is difficult to recommend pharmacopuncture as an evidence-based treatment because of methodological flaws and small sample sizes of the included studies. Further well-designed trials are needed to draw a definitive conclusion.

## 1. Introduction

Pharmacopuncture (herbal acupuncture) is a new form of acupuncture treatment combining acupuncture and the injection of herbal medicine to the acupuncture points (acupoints). In Korea, pharmacopuncture was first officially introduced to the traditional Korean medicine (TKM) community in 1967 by Sang-Cheon Nam. While the conventional acupuncture treatment incorporates the physical stimulation of associated meridians and acupoints, pharmacopuncture adds chemical ingredients from therapeutic herbs with pharmacological effects [1]. In the treatment of pharmacopuncture, the typical constitution and conditions of the individual patient are diagnosed, and specific amounts of herbal extracts are injected into meridians and acupoints, providing the effect of both acupuncture and herbal medicine [1].

The effects of pharmacopuncture can be immediately observed after treatment because herbal extracts are directly absorbed without the need to pass through the gastrointestinal tract. Additionally, both patients with difficulty swallowing and those who refuse to take herbal medicine may profit by receiving pharmacopuncture treatment [1]. The major benefits of pharmacopuncture in comparison to conventional acupuncture are more rapid effects, additional synergistic effects of acupuncture and herbal extracts, and the ease for controlling dosage [2].

Since the 1970s, studies on pharmacopuncture conducted mostly in animals have reported that* Astragali Radix* [3, 4],* Angelica gigas* [4],* Cornus cervi Parvum* [5], and* Sorbus commixta* Hedl. extracts [6] were effective in pain control, immune enhancement, obesity, and arthritis. Since the 2000s, there are a growing number of clinical trials on pharmacopuncture. Currently, numerous types of pharmacopuncture extracts are used. Pharmacopuncture is applied to treat various disorders, most frequently for musculoskeletal conditions. The effectiveness for these disorders has been well studied [1, 7].

This systematic review aims to summarise existing results of randomized controlled trials (RCTs) conducted in Korea to establish the clinical evidence of the safety and efficacy of pharmacopuncture for various conditions.

## 2. Methods

### 2.1. Data Sources and Searches

We searched PubMed, Ovid Medline, and Korean databases, including the Oriental Medicine Advanced Searching Integrated System (OASIS), the Korean Studies Information Service System, RISS4U, Korea Institute of Science and Technology Information, KOREAMED, DBPIA, Korea National Assembly Library, the Journal of Korean Pharmacopuncture Institute, and the Journal of Korean Acupuncture and Moxibustion Medicine Society from inception to December 2014. Reference lists of reviews and relevant articles were examined for additional studies.

The search terms used for PubMed were as follows: (pharmacopuncture*∗*[All Fields] OR “herbal acupuncture” [All Fields] OR “aqua acupuncture” [All Fields] OR aquapuncture*∗*[All Fields] OR “acupoint injection” [All Fields]) AND (“randomized controlled trial” [PT] OR “controlled clinical trial” [PT] OR random*∗*[TIAB] OR placebo [TIAB] OR “drug therapy” [Subheading] OR trial [TIAB] OR groups [TIAB]) NOT Animals [MeSH] NOT Humans [MeSH]. These search terms were slightly modified for other databases. Trials conducted in Korea and published in English or Korean were sought.

### 2.2. Study Selection

#### 2.2.1. Inclusion Criteria

All RCTs evaluating pharmacopuncture treatment on various conditions were considered. Studies enrolling participants who reported any disorder or disease were eligible for inclusion. Hence, we classified each disorder or disease according to the International Statistical Classification of Diseases and Related Health Problems, 10th revision (ICD-10) [8] for the analyses.

Studies which assessed the combined effects of pharmacopuncture plus other interventions (e.g., pharmacopuncture plus acupuncture) were also considered when the identical intervention was administered to both the pharmacopuncture group and the control group.

For control groups, we considered placebo or sham, other interventions, and no interventions. Placebo or sham interventions were injections of normal saline or distilled water into the pharmacopuncture points or nonacupuncture points. Other interventions included acupuncture, herbal/western medicine, cupping, tuina, diet therapy, and physical therapy, including hot pack, transcutaneous electrical nerve stimulation (TENS), interferential current therapy (ICT), ultrasound, massage, and exercise.

There was no restriction on the type of outcome measures, but they had to be relevant to the conditions. All the trials were conducted in Korea.

#### 2.2.2. Exclusion Criteria

Nonrandomized trials, animal or cell studies, literature research, and quasi-RCTs (methods of allocating participants to a treatment group which are not truly random, e.g., hospital record number or alternation, and date of birth) were excluded. Trials including healthy participants were excluded.

We did not include trials testing bee-venom pharmacopuncture or injection of conventional medicine because they did not investigate the chemical effects of herbal medicine. Trials comparing different types of pharmacopuncture were excluded because the efficacy of control intervention could not be determined.

### 2.3. Data Extraction

We reviewed all searched articles to evaluate their eligibility for inclusion. In case of uncertainties, authors were contacted for further information.

After the selection of studies, we extracted the following data from the selected articles: author, year of publication, study design, participants (age, gender), diseases or disorders, pharmacopuncture intervention, control intervention, outcome measures, main results, and adverse events (Table 1). The outcome measures at the end of the treatments were used in data pooling. As for the pharmacopuncture interventions, we summarised each item in terms of the types and methods of pharmacopuncture, regimen, pharmacopuncture points, extraction methods, types of syringe, and amount, depth, and angle of the injection following STRICTA recommendations and the data was modified into the suitable form for trials of pharmacopuncture (Table 2).

### 2.4. Assessment of Risk of Bias (ROB)

We evaluated the ROB for the included studies according to the Cochrane Collaboration's ROB assessment tool [39].

We rated ROB for each item using “Yes (Y, low ROB),” “Unclear (U, uncertain or unknown ROB),” or “No (N, high ROB).” For patient blinding in studies with a placebo control, we assessed the study as having a low ROB when it clearly stated that patients were blinded. For the outcome assessor blinding, we judged that if it was clearly reported that the outcome assessor was blinded or the outcome measure was evaluated by blinded participants only, it was rated as having a low ROB. If the outcome measure was assessed by unblinded participants only, we rated it as having a high ROB. If the outcome measures were mixed with subjective and objective assessments and we could not obviously judge whether the outcome assessor was blinded or not, it was rated as having an unclear ROB. As for the incomplete outcome data reporting it was rated as having a low ROB if the number and reason for attrition were clearly reported in each group and were similar between groups and the percentage of withdrawals and drop-outs did not exceed 20% for short-term and 30% for long-term follow-up [39]. If there was disagreement, it was resolved by discussion with HL and JIK.

### 2.5. Data Analyses

Meta-analysis was performed using the Review Manager software (version 5.2 for Windows; the Nordic Cochrane Centre, Copenhagen, Denmark). We used the mean difference (MD) and 95% confidence intervals (CI) to estimate the effect of an intervention for continuous outcomes using a random-effects model.

If it was impossible to perform statistical pooling, studies were assigned to 1 of 4 categories to classify the result for interpretation. The comparison between two groups was based on the results of original study: (1) positive when the pharmacopuncture group was significantly better than the control group, P; (2) negative when the control group was significantly better than the pharmacopuncture group, N; (3) neutral when there was no significant difference between the groups, NS; and (4) not assessable when the results were complicated or the presented data were insufficient, NA.

To address the heterogeneity among the included studies, the *I* ^2^ test was used. An *I* ^2^ value of 50% or more was considered to be an indicator of a substantial level of heterogeneity [40]. Sensitivity analyses were planned by including studies with low ROBs only or by including pain-related studies with sample sizes ≥40 per arm. We analyzed the trials with low ROBs for randomization and/or allocation concealment only and examined whether the estimate of the intervention effect was affected [41, 42]. For the pain-related trials, studies with ≥40 participants per arm were separately analyzed to see whether any differences in the estimate emerged [43].

## 3. Results

**Figure 1:**
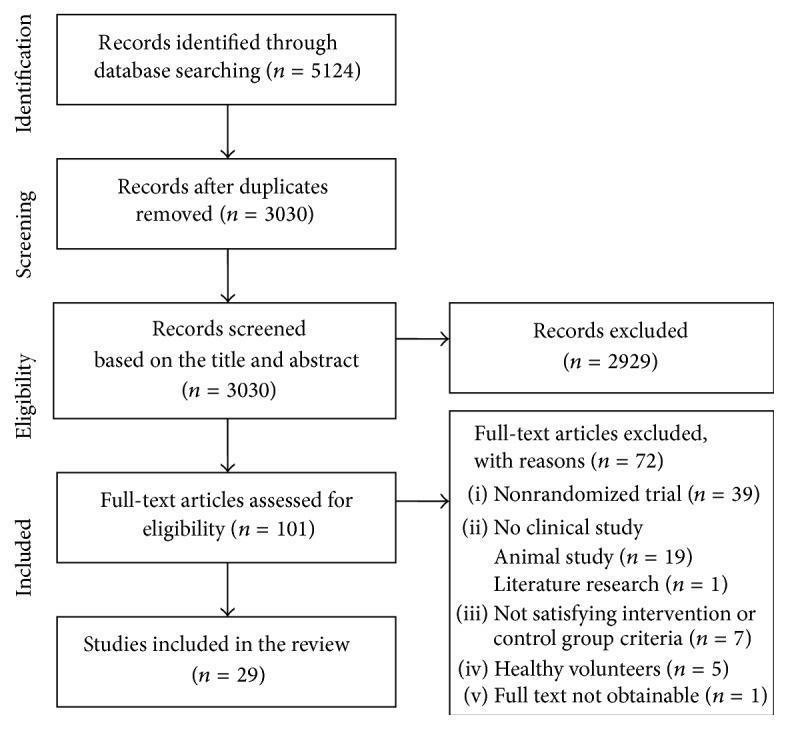
Flow diagram of literature search.

Our search terms yielded 5,124 records: 49 from Ovid Medline or PubMed and 5,075 from domestic databases or relevant journals. After duplicated studies were removed, 3,030 records were screened. Based on the title and abstract, 2,929 records were excluded; 687 articles were inappropriate for the topic of this review; 2,105 were not clinical studies or were nonrandomized trials; and 137 trials did not satisfy the pharmacopuncture or control group criteria. Out of the remaining 101 studies, a total of 29 RCTs (Korean: *n* = 27; English: *n* = 2) were included in our review.  Figure 1 shows a flow diagram of the literature searching as recommended in the Preferred Reporting Items for Systematic Reviews and Meta-Analyses (PRISMA) [44]. Details of the included studies are summarised in Table 1.

### 3.1. Participants

Overall included RCTs (29): data of 1,321 participants were included in the review. The number of participants in each group ranged from 10 to 37 in the pharmacopuncture group and from 9 to 46 in the control group. The median sample sizes per arm were 17 in the pharmacopuncture group and 18 in the control group.

The types of diseases/disorders were very heterogeneous. Thus, we classified them using ICD-10 codes. The most common disorders were diseases of the musculoskeletal system and connective tissue (XIII, *n* = 15). Among them, there were 5 studies each for low back pain [25–27, 31, 33] and cervicalgia [22–24, 28, 29]; two studies were for knee osteoarthritis [30, 34] and shoulder pain [21, 35] each; and one was for ankle sprain [32]. In the category nervous system disease/disorder [12–17] (VI, *n* = 6), three studies of Bell's palsy [13, 14, 17], two of headache [12, 15], and one for carpal tunnel syndrome [16] and leg spasticity of stroke patients [18] were found. The other studies could be classified into the endocrine, nutritional, and metabolic diseases [9–11] (IV, *n* = 3), diseases of the digestive system [19, 20] (XI, *n* = 2), diseases of the circulatory system [18] (IX, *n* = 1), diseases of the genitourinary system [36] (XIV, *n* = 1), and pregnancy, childbirth, and the puerperium [37] (XV, *n* = 1).

### 3.2. Pharmacopuncture Intervention

Details of pharmacopuncture interventions based on the revised STRICTA and modified to suitable patterns for pharmacopuncture are summarised in Table 2 [38].

#### 3.2.1. Types of Pharmacopuncture

When pharmacopuncture was classified by treatment rationale, meridian field pharmacopuncture was practiced in four trials [12, 20, 26, 29], eight-principle pharmacopuncture was administered in 14 studies [13–15, 21–25, 27, 28, 31–33, 35], mono-herbal-type pharmacopuncture was used in nine trials [10, 16–19, 30, 34, 36, 37], and the other two studies could not be classified [9, 11].

The types of pharmacopuncture were highly variable. Out of the 29 included studies, 12 tested monoherbal medicine pharmacopuncture: six studies [17–19, 34, 36, 37] used* Hominis Placenta*; three studies [12, 20, 29] used Carthami-Semen; and* Capsicum frutescens* L. [10], scolopendrid [16], and root bark of* Ulmus davidiana* Planch. (UDP) [30] were used in one study each. The other 17 tested mixed-herbal medicine pharmacopuncture types: five studies [15, 22, 23, 25, 32] used Hwangryunhaedok-tang (*Scutellaria baicalensis*,* Coptis chinensis*,* Phellodendron amurense,* and* Gardenia jasminoides*); four studies [13, 14, 28, 31] used Soyeom pharmacopuncture (*Taraxacum officinale*,* Lonicera japonica*,* Rehmannia glutinosa*,* Forsythia viridissima*,* Coptis chinensis*,* Scutellaria baicalensis*,* Phellodendron amurense,* and* Gardenia jasminoides*); another four studies [21, 24, 33, 35] used Ouhyul pharmacopuncture (*Gardenia jasminoides*,* Corydalis remota*,* Boswellia carteri*,* Commiphora myrrha*,* Prunus persica*,* Paeonia lactiflora*,* Salvia miltiorrhiza,* and* Caesalpinia sappan*); and each used* Ephedra sinica *Stapf and* Aconitum carmichaeli *Debx. [11],* Calculus Bovis*.*Fel Ursi*.*Moschus *(BUM) [26],* Panax ginseng* plus BUM [9], and ShinBaro pharmacopuncture (modification of Chungpa-Juhn (*Saposhnikovia divaricata *Schiskin,* Achyranthes bidentata *Blume,* Acanthopanax sessiliflorum* Seem,* Cibotium barometz *J. Smith,* Glycine max *Merrill, and* Eucommia ulmoides *Oliver)) [27].

#### 3.2.2. Pharmacopuncture Methods

Participants received fixed (i.e., all participants received the same treatment), partially individualized (using a fixed set of points to be given with a set of points to be used flexibly), or individualized pharmacopuncture treatment (each participant received a tailored treatment). Out of the 29 studies, 18 used fixed [9–18, 20, 21, 25, 29, 32, 35–37], 7 studies used partially individualized [19, 22, 23, 26, 28, 30, 34], and the other 4 trials used individualized acupuncture treatments [24, 27, 31, 33].

#### 3.2.3. Treatment Sessions

The number of pharmacopuncture sessions ranged from 3 to 16 over 6 days to 8 weeks.

#### 3.2.4. Pharmacopuncture Points

Regarding pharmacopuncture points used in the studies, 19 studies [11–15, 17–23, 25, 27, 29, 31, 32, 36, 37] used 12 meridian points and/or extra points. Four studies [26, 28, 30, 34] used 12 meridian points, extra points, Ashi points, and tender points together. Two studies [24, 33] used Ashi points only, and one study [35] used 12 meridian points plus Dr. Dong's acupuncture point. Three studies [9, 10, 16] mentioned approximate areas but not the accurate points, such as the abdomen [10], the area between the flexor carpi radialis tendon and the median nerve [16], or the left and right sides of four points inferior to and four points superior to the navel points on the stomach and the spleen and gallbladder meridians [9].

#### 3.2.5. Extraction Methods

As for the extraction methods of pharmacopuncture, 6 studies [11, 13, 15, 21, 27, 30] used distillation of the herbal medicine; three studies [12, 16, 20] used alcohol immersion extraction methods; one study [29] used an extraction method that involved pressing from the herbs; one study [9] used distillation for wild ginseng and alcohol immersion for BUM; and the other 18 studies did not report details about the extraction method. Out of the 11 trials that mentioned extraction methods, 8 studies [9, 11, 13, 15, 16, 21, 27, 29] followed the guidelines of the Korean Pharmacopuncture Institute. Two studies that used Carthami-Semen [12, 20] did not follow the guidelines, in which the pressing extraction method is used to extract Carthami-Semen, but used an alcohol immersion extraction method instead. The other trial [30] used distillation to extract UDP, but there was no guideline for the extraction of UDP.

#### 3.2.6. Types of Injector

In total, 24 studies mentioned the type of injector: 16 studies [13, 15–20, 24, 27–31, 35–37] used 1 mL syringes; one study [10] used a mesogun; and another study [34] reported using a U-100 insulin syringe but did not state the size or gauge of the syringe. The gauge, which indicates the thickness of the needle, was varied. 18 studies stated the gauge: four studies [15, 18, 21, 28] applied a 30-gauge syringe; seven studies [17, 22, 23, 25, 30–32] were done with a 29-gauge syringe; two studies [12, 20] were performed with a 27-gauge syringe; four studies [13, 27, 29, 37] were done using a 26-gauge syringe; and one study [36] used a 26-gauge syringe (CV4) and a 30-gauge syringe (ST36, SP6, and SP9) according to the pharmacopuncture points. Five studies [9, 11, 14, 26, 33] did not mention the gauge.

#### 3.2.7. Amount of Injection

Each amount of injection ranged from 0.05 mL to 1 mL, and the total amount of injection ranged from 0.2 mL to 5 mL. Only one study [30] did not report the amount of injection.

#### 3.2.8. Depth of Injection

Seven studies [16, 18, 20, 27, 29, 30, 36] reported the depth of injection. The depth of injection ranged from 5 to 30 mm according to the pharmacopuncture points.

#### 3.2.9. Angle of Injection

The angle of injection was reported in only three studies: two studies [18, 27] used perpendicular angles, and one study [16] used oblique angle (45 degrees) when injecting into the wrist area.

### 3.3. Control Intervention

In this review, control procedures were classified into four types. First, pharmacopuncture was compared with normal saline [9–12, 15, 17, 20, 21, 30, 35–37] or distilled water injections [18] in 13 studies for blinding. Secondly, pharmacopuncture was tested against tuina manual treatment in three studies [23–25]. Thirdly, three studies adopted acupuncture as a control group [19, 32, 34]. Finally, the comparison of pharmacopuncture plus other interventions and other interventions alone groups was investigated in ten studies [13, 14, 16, 22, 26–29, 31, 33]. Other interventions included acupuncture [13, 14, 16, 22, 26–29, 31, 33], herbal/western medicine [13, 14, 16, 22, 27–29, 31, 33], cupping [28, 31], tuina [27], and physical therapy [13, 14, 16, 27–29, 31, 33] (Table 1).

### 3.4. Outcome Measures

Outcome measures reported in the included studies were very diverse because of the various types of focused diseases. Intensity of discomfort (e.g., measured with the visual analogue scale, the numeric rating scale) was investigated in 20 trials [13–16, 20–33, 35, 37]. All studies focusing on diseases of the musculoskeletal system and connective tissue adhered to it except one [34]. A quality-of-life-related scale was applied in six studies [12, 15, 19, 20, 29, 30]. All trials on Bell's palsy utilized the Yanagihara score [13, 14, 17]. All studies on cervicalgia used a neck disability index [22–24, 28, 29]. Out of the five studies that treated low back pain, three studies were applied using the Oswestry disability index [25–27] (Table 1).

### 3.5. ROB Assessment

The majority of the included trials were assessed as having a high ROB. Details of the ROB assessments are presented in Table 3.

Twelve out of the 25 studies reported adequate methods of sequence generation, such as using a random number table, computer random number generator, randomization code, or coin toss [9, 11–13, 18, 20–22, 24, 27, 29, 36]. Group assignment was adequately concealed in only four trials using sealed opaque envelopes [12, 30] or central allocation [21, 27].

The participant, practitioner, and outcome assessor each were blinded in only two trials [12, 30]. Double-blinding of the participant and practitioner was conducted in two studies [18, 35]. Participant blinding was performed in four trials [10, 11, 17, 37]. The participant and outcome assessor were blinded in three trials [15, 20, 36] as outcome measures were all subjective and assessed by blinded participants in two trials [15, 36], and the other one mentioned that an independent assessor evaluated constipation symptoms [20].

In terms of addressing incomplete outcome data, 13 studies [13–16, 19, 22–24, 27, 29, 31, 34, 36] were assessed as having a low ROB, as they had no missing outcome data. Nine trials [9, 12, 18, 20, 21, 30, 32, 33, 35] had missing outcome data, but the drop-out rate did not exceed 20% for short-term and 30% for long-term follow-up, and the number and reasons for drop-out in each group were similar. Six trials [10, 11, 25, 26, 28, 37] also had missing outcome data, but the drop-out rate exceeded 20% for short-term and 30% for long-term follow-up. The other study [17] had missing outcome data, but we could not calculate the drop-out rate, as the number of participants randomized in each group was not reported.

As for the selective outcome reporting, we could not locate and compare the protocols of any of the included studies. Therefore, we judged the ROB based on the described methods in each study. One study [16] had a high ROB because the authors (Lim et al.) were supposed to report visual analog scale (VAS) in the methods part, but there was no VAS data in results section.

### 3.6. Effects of Pharmacopuncture

The key outcomes from the included studies are provided in Table 1.

Low back pain (*n* = 5) [25–27, 31, 33], cervicalgia (*n* = 5) [22–24, 28, 29], obesity (*n* = 3) [9–11], and Bell's palsy (*n* = 3) [13, 14, 17] were the most actively researched fields using pharmacopuncture intervention.

**Figure 2:**
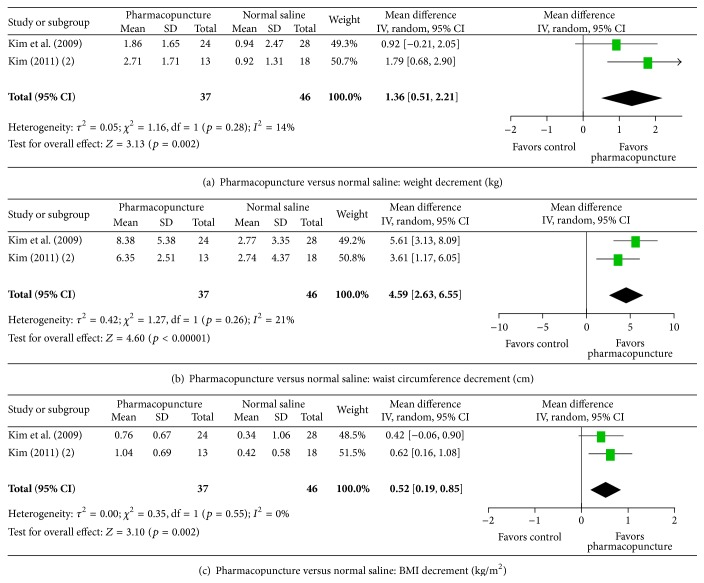
Effect of pharmacopuncture in obesity. BMI: body mass index; CI: confidence intervals; SD: standard deviation.

**Figure 3:**
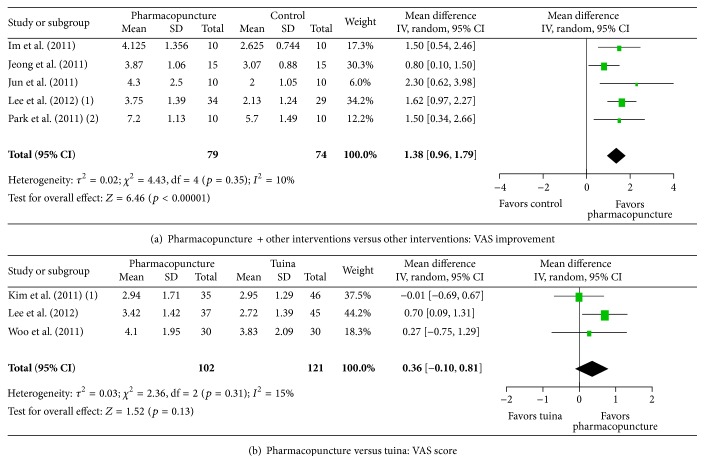
Effects of pharmacopuncture on musculoskeletal conditions. CI: confidence intervals; SD: standard deviation; VAS: visual analog scale.

A total of 10 studies were available for statistical pooling (Figures 2 and 3). As for the other 19 trials in which statistical pooling was impossible because of the substantial heterogeneity of the diseases, types of pharmacopuncture, control groups, or outcome measures, we classified the results into four categories: positive (P), negative (N), neutral (NS), and not assessable (NA).

#### 3.6.1. Effects of Pharmacopuncture in Obesity

Among the three studies on obesity, two studies [10, 11] showed that* Capsicum frutescens* L. or* Ephedra sinica* Stapf-*Aconitum carmichaeli* Debx. pharmacopuncture significantly reduced weight, waist circumference, and BMI compared with the normal saline injection group by 1.36 kg, 4.59 cm, and 0.52 kg/m^2^, respectively, immediately after treatment (Figure 2(a), MD 1.36, 95% CI: 0.51–2.21; Figure 2(b), MD 4.59, 95% CI: 2.63–6.55; Figure 2(c), MD 0.52, 95% CI: 0.19–0.85). There were no significant heterogeneities among the trials (Figure 2(a), *χ* ^2^ = 1.16, degrees of freedom (df) = 1, *p* = 0.28, and *I* ^2^ = 14%; Figure 2(b), *χ* ^2^ = 1.27, df = 1, *p* = 0.26, and *I* ^2^ = 21%; Figure 2(c), *χ* ^2^ = 0.35, df = 1, *p* = 0.55, and *I* ^2^ = 0%). Another study [9] also reported that body weight, waist circumference, and BMI were more reduced than in the normal saline group, but we did not obtain sufficient data for statistical pooling. Thus, the result was not assessable.

#### 3.6.2. Effects of Pharmacopuncture on Musculoskeletal Conditions

In five studies on musculoskeletal diseases [22, 26–29], pharmacopuncture plus other interventions significantly alleviated pain intensity compared with the other interventions only immediately after treatment (Figure 3(a), MD 1.38, 95% CI: 0.96–1.79, and *I* ^2^ = 10%). Three studies that compared pharmacopuncture with tuina manual therapy [23–25] reported that pharmacopuncture was not more effective than tuina in musculoskeletal diseases immediately after treatment (Figure 3(b), MD 0.36, 95% CI: −0.10–0.81, and *I* ^2^ = 15%).

As statistical pooling was impossible in the other seven trials, detailed results were described as follows. Two trials on HNP of the L-spine [31, 33] showed unassessable effects of Soyeom and Ouhyul pharmacopuncture. Of the two studies on osteoarthritis of the knee, one study [30] contrasted root bark of UDP pharmacopuncture with normal saline injection, and the effect was not assessable. Another study [34] that compared* Hominis Placenta* Pharmacopuncture with acupuncture showed no significant difference between the groups. In shoulder pain caused by stroke [21, 35], the effect of pharmacopuncture was not assessable. For acute ankle sprain [32], acupuncture improved the symptoms better than Hwangryunhaedok-tang pharmacopuncture after 9–12 days of treatments; in other words, they reported a negative effect of pharmacopuncture.

#### 3.6.3. Effects of Pharmacopuncture on Diseases of the Nervous System

The results of pharmacopuncture were composited for each disease. For headache, both Carthami-Semen and Hwangryunhaedok-tang pharmacopuncture improved symptoms compared with normal saline injection [12, 15]. For Bell's palsy, the effect of* Hominis Placenta* Pharmacopuncture was significantly better than normal saline injection [17]. However, for the postauricular pain that accompanies Bell's palsy, the effect of Soyeom pharmacopuncture was not assessable. It may be due to the fact that the pain intensity or duration of pain decreased significantly, while the Yanagihara score did not show a significant difference between the groups [13, 14]. One study on scolopendrid pharmacopuncture treatment did not show additional effects on pain intensity in carpal tunnel syndrome [16].

#### 3.6.4. Effects of Pharmacopuncture on Diseases of the Circulatory System

The effect of* Hominis Placenta* Pharmacopuncture compared with distilled water injection was not assessable in leg spasticity of stroke patients due to the complexity of the results. For modified Ashworth scale (MAS), H-reflex/M-response ratio (H/M ratio), and Berge balance scale (BBS), there were no significant differences between groups, while the time up and go (TUG) in pharmacopuncture group was significantly lower than in distilled water injection group [18].

#### 3.6.5. Effects of Pharmacopuncture on Diseases of the Digestive System

One study of* Hominis Placenta* Pharmacopuncture had a similar effect to acupuncture in dyspepsia [19]. The effect of Carthami-Semen pharmacopuncture on chronic constipation was not assessable [20].

#### 3.6.6. Effects of Pharmacopuncture on Diseases of the Genitourinary System

One study on* Hominis Placenta* Pharmacopuncture for dysmenorrhea showed a similar effect to normal saline injection [36].

#### 3.6.7. Effects of Pharmacopuncture on Pregnancy, Childbirth, and the Puerperium

*Hominis Placenta* Pharmacopuncture had complicated results compared with normal saline injection [37]. There were no significant differences between groups in VAS for heating feeling, sweet during movement and sleeping, and complete blood cell (CBC) count. For thirst,* Hominis Placenta* Pharmacopuncture group showed significant higher VAS than normal saline injection group before the treatment (*p* = 0.023). After treatment, two groups reported similar thirst symptom (*p* = 0.510) without correcting the baseline value. Therefore, we could not assess the results.

### 3.7. Adverse Events (AEs)

Only five studies reported AEs. In the study by Seo [21], they compared Ouhyul pharmacopuncture with normal saline and reported general pain in the Ouhyul group and transient local site pain or fatigue in the normal saline group. Kim [10] compared* Capsicum frutescens* L. with normal saline and reported moderate AEs related to anesthesia cream or pharmacopuncture (4 in the* Capsicum frutescens* L. group, 2 in the normal saline group). In each of the two studies by Park et al. [12, 20], they compared Carthami-Semen with normal saline and reported mild AEs, such as pain during the injection, ecchymosis, and redness in the Carthami-Semen group and moderate pain during the injection in the normal saline group. One study by Kim et al. [30] compared UDP with normal saline and reported mild AEs, such as nausea and itching in the UDP group and slight dizziness in the normal saline group. These AEs disappeared in a short time without specific treatment, and no serious AEs were reported. Another two studies by Jun et al. [27] and Kim et al. [37] reported that AEs did not occur, and the other 22 trials [9, 11, 13–19, 22–26, 28, 29, 31–36] did not mention AEs.

### 3.8. Sensitivity Analyses

We performed sensitivity analyses by excluding studies with predefined less desirable characteristics, and the results from the musculoskeletal studies were robust.

#### 3.8.1. ROB

When the analyses were limited to two studies with a low ROB for random sequence generation and/or allocation concealment [27, 29], pharmacopuncture had additional benefits in terms of pain relief in musculoskeletal diseases immediately after treatments (MD 1.76, 95% CI: 0.80–2.71, *I* ^2^ = 0%). One study with adequate random sequence generation and/or allocation concealment did not provide enough information; thus, the effect of pharmacopuncture compared with normal saline could not be assessed in shoulder pain caused by stroke [21].

#### 3.8.2. Sample Size

There was no study with ≥40 participants per arm.

## 4. Discussion

Our review on pharmacopuncture aimed to establish the evidence of pharmacopuncture treatment of any disease. The analyses of two trials on obesity [10, 11] demonstrated a significant benefit from 5 to 8 weeks of pharmacopuncture treatments compared with normal saline injection. The analyses of five trials on musculoskeletal diseases [22, 26–29] represented a significant effect from 7 to 30 days of combined treatment of pharmacopuncture with other interventions (e.g., acupuncture, herb medicine, tuina, and physical therapy) compared with other interventions only. However, these analyses were based on small studies and other interventions used in these trials were varied; thus cautious interpretation is needed. In the musculoskeletal diseases, pharmacopuncture's benefits were maintained by limiting the analyses to studies with a low ROB for randomization and/or allocation concealment [27, 29], which means that they presented robust evidence for the treatment of musculoskeletal diseases. However, the number of participants in these studies was too small (less than 10 per arm), so careful interpretation is required. Pharmacopuncture does not appear to be associated with serious AEs, but the evidence is limited.

Most of the included studies had methodological weaknesses. Thirteen out of 25 studies [9, 11–13, 18, 20–22, 24, 27, 29, 30, 36] had low ROBs for adequate randomization and/or allocation concealment. Among them, only three studies [12, 21, 27] had both appropriate randomization and allocation concealment. It is well known that inadequate allocation concealment/random sequence generation leads to the overestimation of treatment effects [41, 42], and unconcealed allocation is the most important source of bias in RCTs [45]. When we limited our analyses to the studies rated as having low ROBs for randomization/allocation concealment, pharmacopuncture's benefit was maintained.

There were some limitations in this review. Our review only included trials conducted in Korea and published in Korean or English. Therefore, we could not necessarily remove a potential language bias. Egger et al. [46] reported that studies published in non-English languages or studies published in journals that are not indexed in Medline are likely to increase the effect estimates, and this may have relevance to this review. In addition, pharmacopuncture, an acupuncture-related intervention, may be highly culture specific. According to the 2007 National Health Interview Survey (NHIS) data, only 6.5% of the Americans reported ever receiving acupuncture [47]. Upchurch et al. also reported that there was significant difference in the use of acupuncture by ethnicity and race. Asian women reported the highest usage [48]. Thus, further research is necessary to determine whether the interventions are applicable and acceptable in other countries.

The included trials were mostly of poor quality; thus, the reported data are likely to be overestimated. In addition, most of the included studies were small. Median sample sizes per arm were 17 in the pharmacopuncture group and 18 in the control group. The effect size of small studies may have been inflated due to poor methodological design and conduct [49]. Moore et al. [43] reported in a simulation study that at least 40 participants per arm are required to obtain clinically relevant results in trials of pain; however, there were no studies with ≥40 participants per arm, and we could not analyze it separately.

The efficacy of the treatment used for the control group, such as acupuncture, herbal medicine, and tuina manual therapy, was not yet established; therefore, we could not attribute “positive” results solely to the effectiveness of pharmacopuncture. Additionally, clinically meaningful information on follow-up results was sparse in the majority of the included trials. Therefore, the available evidence prevented us from further examining how long its benefit was maintained.

As we included all conditions/diseases, the focus of our review may seem blurred. However, this review provides an overview of the entire primary pharmacopuncture researches conducted in Korea. The results help to set priorities and directions for future research on pharmacopuncture.

Although this review represented the applicability of pharmacopuncture, the standardisation of pharmacopuncture intervention was not performed. Thus, in the future, it is absolutely necessary to standardise it to apply pharmacopuncture in routine clinical practice. The degree of pharmacopuncture stimulation could be influenced by the following factors: (1) pharmacopuncture types; (2) concentration and extraction methods of pharmacopuncture; (3) amount, depth, and angle of injection; (4) syringe types, including thickness and length; (5) pharmacopuncture points; and (6) number of sessions based on the STRICTA guidelines [38]. Currently, the Korean Pharmacopuncture Institute suggests guidelines for pharmacopuncture treatment. The classification of pharmacopuncture, such as meridian field, eight-principle, or monoherbal medicine-type, is determined by the diagnosis of patient's conditions. The total amount of injections depends on the severity of the disorder, the age of the patient, the injecting area, and the characteristics and concentration of the pharmacopuncture extract. Using various types of injectors or syringes depends on the type of pharmacopuncture, its dosage, the area of the body part, and the depth of the injection. Syringe needles are generally between 26 and 32 gauges. Different needles are utilized for different uses.

However, the standardisation of these factors has yet to be completed, and there is no firmly established research method for pharmacopuncture studies; therefore, pharmacopuncture interventions of the included trials were very heterogeneous. In addition, future studies should include not only a test of the efficacy and safety of pharmacopuncture but also an examination of the validity of the intervention based on the standardised guidelines.

## 5. Conclusions

The results of this review demonstrate the effectiveness of pharmacopuncture for the treatment of obesity and musculoskeletal diseases compared with normal saline injections and other interventions, respectively; however, given the methodological flaws and small sample sizes, the available evidence is insufficient to recommend pharmacopuncture as an evidence-based treatment option. In the future, the standardisation of pharmacopuncture intervention and the adequate reporting of pharmacopuncture intervention in accordance with STRICTA guidelines are needed.

## Figures and Tables

**Table 1:** Characteristics of the included studies.

Author, year	Design	Types of disease	Sample size (M/F)	Pharmacopuncture group (No. of participants analyzed/randomized)	Control group (No. of participants analyzed/ randomized)	Outcome measures	Main results	AE
*(IV) Endocrine, nutritional, and metabolic diseases (n = 3)*								

Lim, 2013 [9]	Parallel 2 arms	Abdominal obesity	28 (0/28)	(A) Wild ginseng complex pharmacopuncture (14/15)	(B) NSP (14/15)	(1) Anthropometry ① BMI, ② weight, ③ WC ④ HC, and ⑤ WHR (2) Blood test ① TC, ② TG, ③ HDL cholesterol, ④ LDL cholesterol, ⑤ CRP, and ⑥ AST, ALT, *γ*-GT, BUN, and creatinine (3) Body composition (BFM, BFP, FFM, and BMR) (4) Abdominal fat (TFA, SFA, VFA, and VSR)	(1) ①, ②, and ③ Positive^a^ ④, ⑤ NS (2) ①, ②, ③, ⑤, and ⑥ NS ④ Positive^a^ (3) NS (4) NS	n.r.

Kim, 2011 (2) [10]	Parallel 2 arms	Abdominal obesity	31 (0/31)	(A) *Capsicum frutescens* L. pharmacopuncture + diet therapy + exercise (13/20)	(B) NSP + diet therapy + exercise (18/20, in case of outcome measures 1, 2, and 4; 16/20, in case of outcome measure 3)	(1) Anthropometry ① BMI, ② WC ③ weight, and ④ WHR (2) Abdominal fat (TFA, VFA) (3) Energy expenditure (4) Questionnaires	(1) ①, ② Positive^a^ ③, ④ Positive^b^ (2) Positive^b^ (3) NA (4) NS	Moderate AEs related with anesthesia cream or pharmacopuncture (4 in group (A), 2 in group (B))

Kim et al., 2009 [11]	Parallel 2 arms	Obesity	52 (8/44)	(A) EAP + diet therapy + EA + HM + exercise (24/35)	(B) NSP + diet therapy + EA + HM + exercise (28/35)	(1) Weight (2) BMI (3) Waist	(1) Positive^a^ (2) Positive^a^ (3) Positive^c^	n.r.

*(VI) Diseases of the nervous systems (n = 6)*								

Park et al., 2011 (1) [12]	Parallel 2 arms	Chronic headache	35 (5/30)	(A) CS pharmacopuncture (17/20)	(B) NSP (18/20)	(1) HIT (2) No. of headache-free days (3) SF-36	(1) Positive^a^ (2) Positive^a^ (3) Positive^a^	Injection-site pain (2 in group (A), 3 in group (B)), ecchymosis (2 in group (A))

Shin et al., 2009 [13]	Parallel 2 arms	Postauricular pain accompanied with Bell's palsy	30 (15/15)	(A) Soyeom pharmacopuncture + A + HM + PT (SSP, massage, exercise, HP, and ICT) (15/15)	(B) A + HM + PT (SSP, massage, exercise, HP, and ICT) (15/15)	(1) VAS (2) Duration of pain (d) (3) Yanagihara score	(1) Positive^a^ (2) Positive^a^ (3) NS	n.r.

Choi et al., 2009 [14]	Parallel 2 arms	Postauricular pain accompanied with Bell's palsy	30 (14/16)	(A) Soyeom pharmacopuncture + A + HM + PT (SSP, massage, exercise, ICT, and negative) (15/15)	(B) A + HM + PT (SSP, massage, exercise, ICT, and negative) (15/15)	(1) VAS (2) Yanagihara score	(1) Positive^c^ (2) NS	n.r.

Kim et al., 2006 [15]	Parallel 2 arms	Functional headache	26 (11/15)	(A) HHT pharmacopuncture (13/13)	(B) NSP (13/13)	(1) VAS (2) BPI	(1) Positive^a^ (2) Positive^a^	n.r.

Lim et al., 2005 [16]	Parallel 2 arms	Carpal tunnel syndrome	40 (7/33)	(A) Scolopendrid pharmacopuncture + A + EA + HM + PT (PB, ultrasound, HP, microwave, ICT, and SSP) (20/20)	(B) A + EA + HM + PT (PB, ultrasound, HP, microwave, ICT, and SSP) (20/20)	(1) VAS (2) PRGA based on symptoms and VAS (excellent/good/ fair/poor)	(1) n.r. (2) NS	n.r.

Lee et al., 2005 [17]	Parallel 2 arms	Bell's palsy	44 (21/23)	(A) HPP + A + HM + WM + PT (EST, IR, HP, massage, and exercise) (23/23)	(B) NSP + A + HM + WM + PT (EST, IR, HP, massage, and exercise) (21/21)	Yanagihara score	Positive^a^	n.r.

*(IX) Diseases of the circulatory system (n = 1)*								

Noh et al., 2009 [18]	Parallel 2 arms	Leg spasticity of stroke patients	20 (8/12)	(A) HPP + A (11/11, in case of outcome measures 1, 3, and 4; 10/11, in case of outcome measure 2)	(B) Distilled water pharmacopuncture + A (9/9)	(1) MAS (2) H/M ratio (3) BBS (4) TUG	(1) NS (2) NS (3) NS (4) Positive^a^	n.r.

*(XI) Diseases of the digestive system (n = 2)*								

Lee, 2013 [19]	Parallel 2 arms	Dyspepsia	60 (16/44)	(A) HPP + HM (30/30)	(B) A + HM (30/30)	NDI-K	NS	n.r.

Park et al., 2008 [20]	Parallel 2 arms	Chronic constipation	21 (5/16)	(A) CS pharmacopuncture (11/12)	(B) NSP (10/12)	(1) Defecation frequency, consistency, and ease of evacuation (2) VAS (3) QOL (4) HRV	(1) Significant difference in (A)^a^; N in (B) (2) Significant difference in (A)^a^; N in (B) (3) N (4) Significant improvement of LF/HF ratio in (A) after 2-week follow-up^a^	Mild AEs such as ecchymosis, pain during injection, and redness in group (A) Moderate pain during injection in group (B)

*(XIII) Diseases of the musculoskeletal system and connective tissue (n = 15)*								

Seo, 2013 [21]	Parallel 2 arms	Shoulder pain caused by stroke	24 (10/14)	(A) Ouhyul pharmacopuncture + other treatments (13/16)	(B) NSP + other treatments (11/13)	(1) NRS (2) PROM (3) FMMA	(1) NA (2) NS (3) NS	General pain (1 in group (A)), transient local site pain (1 in group (B)), and fatigue (1 in group (B))

Lee et al., 2012 (1) [22]	Parallel 3 arms	Cervicalgia caused by TA	87 (42/45)	(A) HHT pharmacopuncture + tuina + A + HM + IR (34/34)	(B) Tuina + A + HM + IR (29/29) (C) HHT pharmacopuncture + A + HM + IR (24/24)	(1) VAS (2) NDI	(1) Positive^b^ (2) Positive^a^	n.r.

Lee et al., 2012 (2) [23]	Parallel 2 arms	Cervicalgia caused by TA	82 (40/42)	(A) HHT pharmacopuncture + A + HM (37/37)	(B) Tuina + A + HM (45/45)	(1) VAS (2) NDI	(1) NS (2) NS	n.r.

Woo et al., 2011 [24]	Parallel 2 arms	Cervicalgia caused by TA	60 (28/32)	(A) Ouhyul pharmacopuncture + A + IR (30/30)	(B) Tuina + A + IR (30/30)	(1) VAS (2) NDI	(1) NS (2) NS	n.r.

Kim et al., 2011 (1) [25]	Parallel 2 arms	LBP caused by TA	81 (44/37)	(A) HHT pharmacopuncture + A + HM (35/49)	(B) Tuina + A + HM (46/49)	(1) ODI (2) VAS	(1) NS (2) NA	n.r.

Jeong et al., 2011 [26]	Parallel 2 arms	Acute LBP	30 (21/9)	(A) BUM pharmacopuncture + A (15/21)	(B) A (15/21)	(1) VAS (2) ODI	(1) Positive^a^ (2) NS	n.r.

Jun et al., 2011 [27]	Parallel 2 arms	HNP of the L-spine	20 (9/11)	(A) ShinBaro pharmacopuncture + A + CCP + HM + tuina + PT (ICT, TENS, microwave, and HP) (10/10)	(B) A + CCP + HM + tuina + PT (ICT, TENS, microwave, and HP) (10/10)	(1) NRS (2) ODI	(1) ① LBP decrement: positive^a^ ② Sciatica decrement: N (2) NS	None

Im et al., 2011 [28]	Parallel 2 arms	Acute cervicalgia by TA	20 (7/13)	(A) Soyeom pharmacopuncture + A + HM + cupping + PT (ICT, ultrasound, and HP) (10/13)	(B) A + HM + cupping + PT (ICT, ultrasound, and HP) (10/13)	(1) VAS (2) NDI (3) DITI	(1) Positive^a^ (2) NS (3) NS	n.r.

Park et al., 2011 (2) [29]	Parallel 2 arms	Cervicalgia	20 (0/20)	(A) Carthami-Flos pharmacopuncture + A + HM + PT (HP, TENS, and ICT) (10/10)	(B) A + HM + PT (HP, TENS, and ICT) (10/10)	(1) VAS (2) NDI (3) MENQOL	(1) Positive^a^ (2) Positive^b^ (3) Positive^c^	n.r.

Kim et al., 2010 (1) [30]	Parallel 2 arms	OA (knee)	53 (9/44)	(A) Root bark of UDP pharmacopuncture (29/30)	(B) NSP (24/30)	(1) VAS (2) WOMAC (pain/total score) (3) KHAQ (4) SF-36	(1) NA (2) NS (3) NS (4) NS	Nausea, itching (1 in group (A)), and dizziness (1 in group (B))

Song et al., 2009 [31]	Parallel 2 arms	HNP of the L-spine	30 (15/15)	(A) Soyeom pharmacopuncture + A + HM + PT (TENS, HP, and ICT) + wet cupping (15/15)	(B) A + HM + PT (TENS, HP, and ICT) + wet cupping (15/15)	(1) VAS (2) SLRT	(1) Positive^b^ (2) NS	n.r.

Kang et al., 2008 [32]	Parallel 3 arms	Acute ankle sprain	52 (17/35)	(A) HHT pharmacopuncture (17/20)	(B) A (17/20) (C) BVP (18/20)	(1) NRS (2) AHS	(1) Negative^b^ (2) Negative^a^	n.r.

Lee et al., 2007 [33]	Parallel 3 arms	HNP of the L-spine	60 (28/32)	(A) Ouhyul pharmacopuncture + A + HM + PT (HP, ICT, TENS, and negative) (20/20, in case of outcome measure 1, 2; 6/20, in case of outcome measure 3)	(B) A + HM + PT (HP, ICT, TENS, and negative) (20/20, in case of outcome measures 1, 2; 8/20, in case of outcome measure 3) (C) BVP + A + HM + PT (HP, ICT, TENS, and negative) (20/20, in case of outcome measures 1, 2; 9/20, in case of outcome measure 3)	(1) VAS (2) PRGA based on symptoms and physical examination (excellent/good/ fair/poor) (3) SLRT	(1) Positive^a^ (2) NA (3) NS	n.r.

Park et al., 2006 [34]	Parallel 2 arms	OA (knee)	60 (35/25)	(A) HPP + PT (HP, ultrasound massage, ICT, FES, and exercise) (30/30)	(B) A + PT (HP, ultrasound massage, ICT, FES, and exercise) (30/30)	(1) Lysholm score (2) Nine-point scale	(1) NS (2) NS	n.r.

Bae and Park, 2004 [35]	Parallel 2 arms	Shoulder pain caused by stroke	41 (19/22)	(A) Ouhyul pharmacopuncture + A + HM + PT (21/21)	(B) NSP + A + HM + PT (20/22)	(1) MBI (2) Weakness grade (3) NIHSS (4) AI (5) VAS	(1) NS (2) Significant difference in (B)^a^ (3) NS (4) NS (5) NS	n.r.

*(XIV) Diseases of the genitourinary system (n = 1)*								

Kim et al., 2008 [36]	Parallel 2 arms	Dysmenorrhea	49 (0/49)	(A) HPP (25/25)	(B) NSP (24/24)	(1) MMP (2) MSSL	(1) NS (2) NS	n.r.

*(XV) Pregnancy, childbirth, and the puerperium (n = 1)*								

Kim et al., 2010 (2) [37]	Parallel 2 arms	Postpartum women's heat feeling, sweat, and thirst	25 (0/25)	(A) HPP + A + HM (13/16)	(B) NSP + A + HM (12/16)	(1) VAS ① Heating feeling ② Thirst ③ Sweet in movement ④ Sweet during sleeping (2) CBC (3) 7-zone-diagnostic system (4) HRV	(1) ① NS ② NA ③ NS ④ NS (2) NS (3) NS (4) Positive^c^	None

Disease classification according to the ICD-10 code. Data are expressed as mean ± SD unless stated otherwise.

^a^ *p* < 0.05; ^b^ *p* < 0.01; ^c^ *p* < 0.001.

A: acupuncture; AE: adverse event; AHS: ankle-hindfoot scale; AI: activity index; BBS: Berg balance scale; BFM: body fat mass; BFP: body fat percentage; BMI: body mass index; BMR: basal metabolic rate; BPI: brief pain inventory (general activity, mood, enjoyment of life, relations with other people, and sleep); BUM: *Calculus Bovis*.*Fel Ursi*.*Moschus*; BVP: bee-venom pharmacopuncture; CBC: complete blood cell count; CCP: *Coptis chinensis* pharmacopuncture; CS: Carthami-Semen; d: days; DITI: digital infrared thermographic imaging; EA: electroacupuncture; EAP: *Ephedra sinica* Stapf-*Aconitum carmichaeli* Debx. pharmacopuncture; EST: electrical stimulation therapy; F: female; FES: functional electrical stimulation; FFM: fat-free mass; FMMA: Fugl-Meyer motor assessment; HC: hip circumference; HHT: Hwangryunhaedok-tang; HIT: headache impact test; HM: herb medicine; H/M ratio: H-reflex/M-response ration; HNP of L-spine: herniation of nucleus pulposus of lumbar spine; HP: hot pack; HPP: *Hominis Placenta* Pharmacopuncture; HRV: heart rate variability; ICT: interferential current therapy; IR: infraradiation; KHAQ: Korean Health Assessment Questionnaire; LBP: low back pain; M: male; MAS: modified Ashworth scale; MBI: modified Barthel index; MENQOL: menopause-specific quality of life questionnaire; MMP: measure of menstrual pain; MPQ-SF: McGill Pain Questionnaire-Short Form; MSSL: menstrual symptom severity list; N: no significant difference before and after treatment; NA: not assessable; NDI: neck disability index; NDI-K: Nepean Dyspepsia Index-Korean; negative: (B) significantly better than (A); NIHSS: National Institutes of Health Stroke Scale; No.: number; n.r.: not reported; NRS: numerical rating scale; NS: neutral (no significant difference between groups); NSP: normal saline pharmacopuncture; OA: osteoarthritis; ODI: Oswestry disability index; PB: paraffin bath; positive: (A) significantly better than (B); PRGA: patient-reported global assessment; PROM: painless passive range of motion; PT: physical therapy; QOL: quality of life; SFA: subcutaneous fat area; SF-36: short form 36 health survey; SLRT: straight leg raising test; SSP: silver spike point; TA: traffic accident; TC: total cholesterol; TENS: transcutaneous electrical nerve stimulation; TFA: total fat area; TG: triglyceride; TUG: time up and go; UDP: *Ulmus davidiana* Planch.; VAS: visual analog scale; VFA: visceral fat area; VSR: visceral VFA/SFA ratio; WC: waist circumference; WHR: waist-hip ratio; wks: weeks; WM: western medicine; WOMAC: Western Ontario and McMaster Universities.

**Table 2:** Characteristics of pharmacopuncture interventions in the included studies.

Author, year	Types and methods of pharmacopuncture^*∗*^	Regimen	Pharmacopuncture points^†^	Extraction method (does it follow guideline?^‡^)	Types of syringe	Amount of injection	Depth of injection	Angle of injection	Cointerventions
*Meridian field pharmacopuncture (n = 4)*									

Park et al., 2011 (1) [12]	CS, fixed	8 sessions (twice a wk for 4 wks)	Bilateral GB20, GB21, and EX-HN5	Alcohol immersion (N)	27 G	0.1 mL each	n.r.	n.r.	None

Jeong et al., 2011 [26]	BUM, partially individualized	3 sessions (for 7 d)	Bilateral BL23, BL25, and BL26 + tender points	n.r.	n.r.	0.05 mL × 10 (total 0.5 mL)	n.r.	n.r.	A

Park et al., 2011 (2) [29]	Carthami-Flos, fixed	15 sessions (once per 2 d for 30 d)	Bilateral GB20, GB21	Pressing (Y)	1.0 mL syringe, 26 G	0.05 mL × 4 (total 0.2 mL)	10–30 mm	n.r.	A + HM + PT (HP, TENS, and ICT)

Park et al., 2008 [20]	CS, fixed	8 sessions (twice a wk for 4 wks)	ST25, ST27, BL52, and CV6	Alcohol immersion (N)	1.0 mL syringe, 27 G	0.1 mL × 7 (total 0.7 mL)	0.5–1 inch	n.r.	None

*Eight-principle pharmacopuncture (n = 14)*									

Seo, 2013 [21]	Ouhyul, fixed	6 sessions (3 times a wk for 2 wks)	Unilateral LI15, TE14, GB21, SI11, and SI12	Distillation (Y)	30 G	0.1 mL × 5 (total 0.5 mL)	n.r.	n.r.	Other treatments (the type is not mentioned)

Lee et al., 2012 (1) [22]	HHT, partially individualized	8 sessions (twice a wk for 4 wks)	GV16, GB20, GB21, and so forth	n.r.	29 G	Total 1 mL	n.r.	n.r.	A + HM + IR

Lee et al., 2012 (2) [23]	HHT, partially individualized	8 sessions (twice a wk for 4 wks)	GV16, GB20, GB21, and so forth	n.r.	29 G	Total 1 mL	n.r.	n.r.	A + HM

Woo et al., 2011 [24]	Ouhyul, individualized	4 sessions (twice a wk for 2 wks)	Ashi points (neck)	n.r.	1.0 mL syringe	Total 1 mL	n.r.	n.r.	A + IR

Kim et al., 2011 (1) [25]	HHT, fixed	8 sessions (twice a wk for 4 wks)	Bilateral BL23, BL25, GV3, GB30, and so forth	n.r.	29 G	Total 1 mL	n.r.	n.r.	A + HM

Jun et al., 2011 [27]	ShinBaro, individualized	14 sessions (daily for 2 wks)	EX-B2 in the most severe level of disc herniation	Distillation (Y)	1.0 mL syringe, 26 G	1 mL × 2 (total 2 mL)	Intramuscular (3 cm)	Perpendicular	A + CCP + HM + tuina + PT (ICT, TENS, microwave, and HP)

Im et al., 2011 [28]	Soyeom, partially individualized	5 sessions (once per 2 d for 10 d)	Ashi points (neck) + tender points + bilateral GB20, GB21, and GV14	n.r.	1.0 mL syringe, 30 G	0.05–0.1 mL each (total 0.8 mL)	n.r.	n.r.	A + HM + cupping + PT (ICT, ultrasound, and HP)

Song et al., 2009 [31]	Soyeom, individualized	3 sessions (once per 2 d for 7 d)	EX-B2 in the level of disc herniation	n.r.	1.0 mL syringe, 29 G	1 mL × 2 (total 2 mL)	n.r.	n.r.	A + HM + PT (TENS, HP, and ICT) + wet cupping

Shin et al., 2009 [13]	Soyeom, fixed	3 sessions (once per 2 d for 6 d)	TE17 on the affected side	Distillation (Y)	1.0 mL syringe, 26 G	Total 0.6–0.8 mL	n.r.	n.r.	A + HM + PT (SSP, massage, exercise, HP, and ICT)

Choi et al., 2009 [14]	Soyeom, fixed	n.r.	TE17 on the affected side	n.r.	n.r.	Total 0.4 mL	n.r.	n.r.	A + HM + PT (SSP, massage, exercise, ICT, and negative)

Kang et al., 2008 [32]	HHT, fixed	3 sessions (once per 3-4 d × 3)	GB39, GB40, GB41, BL60, BL62, and ST36	n.r.	29 G	0.1 mL × 6 (total 0.6 mL)	n.r.	n.r.	None

Lee et al., 2007 [33]	Ouhyul, individualized	for 9 d	Ashi points (lumbar)	n.r.	n.r.	0.6 mL each	n.r.	n.r.	A + HM + PT (HP, ICT, TENS, and negative)

Kim et al., 2006 [15]	HHT, fixed	4 sessions (once per 2 d for 8 d)	Bilateral GB20, GB21, and LI4	Distillation (Y)	1.0 mL syringe, 30 G	0.1 mL × 6 (total 0.6 mL)	n.r.	n.r.	None

Bae and Park, 2004 [35]	Ouhyul, fixed	3 sessions (once per 2 d for 6 d)	SI10, LI15, TE14, and GB21 + Gyun-joong (Master Dong's acupuncture point)	n.r.	1.0 mL syringe	0.05–0.1 mL × 5 (total 0.25–0.5 mL)	n.r.	n.r.	A + HM + PT

*Monotype pharmacopuncture (n = 9)*									

Lee, 2013 [19]	HPP, partially individualized	6 sessions (3 times a wk for 2 wks)	ST19, ST25, ST27, BL18, BL20, BL21, BL23, and so forth	n.r.	1.0 mL syringe, 0.3 × 8 mm	0.05 mL each (total 0.8 mL)	n.r.	n.r.	HM

Kim, 2011 (2) [10]	*Capsicum frutescens* L., fixed	16 sessions (twice a wk for 8 wks)	Abdomen	n.r.	Mesogun	0.05 mL × 60 (total 3 mL)	n.r.	n.r.	Diet therapy + exercise

Kim et al., 2010 (1) [30]	Root bark of UDP, partially individualized	12 sessions (twice a wk for 6 wks)	ST35, EX-LE5, EX-LE2, and Ashi points on the affected side	Distillation (no guideline)	1.0 mL syringe, 29 G	n.r.	5–15 mm	n.r.	None

Kim et al., 2010 (2) [37]	HPP, fixed	5 sessions (postpartum 6, 8, 10, 12, and 14 d)	CV4, bilateral BL23	n.r.	1.0 mL syringe, 26 G	0.4 mL (CV4), 0.3 mL (BL23) (total 1 mL)	n.r.	n.r.	A + HM

Noh et al., 2009 [18]	HPP, fixed	15 sessions (5 times a wk for 3 wks)	ST36, GB34, BL55, BL56, and BL57	n.r.	1.0 mL syringe, 30 G	0.4 mL × 5 (total 2 mL)	10 mm	Perpendicular	A

Kim et al., 2008 [36]	HPP, fixed	5 sessions^§^	CV4, bilateral ST36, SP9, and SP6	n.r.	1.0 mL syringe, 26 G (CV4), 30 G (bilateral ST36, SP9, and SP6)	1 mL (CV4), 1 mL (bilateral ST36, SP9, and SP6) (total 2 mL)	Equal to needle length	n.r.	None

Park et al., 2006 [34]	HPP, partially individualized	6–9 sessions (2-3 times per wk for 3 wks)	BL23, ST35, EX-LE4, GB34, SP10, and ST34 + Ashi points	n.r.	U-100 insulin syringe	0.1 mL each	n.r.	n.r.	PT (HP, ultrasound massage, ICT, FES, and exercise)

Lim et al., 2005 [16]	Scolopendrid, fixed	8–12 sessions (2-3 times per wk for 4 wks)	Between flexor carpi radialis tendon and median nerve	Alcohol immersion (Y)	1.0 mL syringe	1 mL each	Subcutaneous (flexor retinaculum)	45 degrees	A + EA + HM + PT (PB, ultrasound, HP, microwave, ICT, and SSP)

Lee et al., 2005 [17]	HPP, fixed	3 times per wk (during hospitalization); twice per wk (during the period they visited the clinic) for 5 wks	GB14, SI18, ST4, ST6, TE17, and TE23 on the affected side	n.r.	1.0 mL syringe, 29 G	0.05 mL × 6 (total 0.3 mL)	n.r.	n.r.	A + HM + WM + PT (EST, IR, HP, massage, and exercise)

*Others (n = 2)*									

Lim, 2013 [9]	Wild ginseng + BUM, fixed	10 sessions (twice a wk for 5 wks)	Left and right sides of four points inferior to and four points superior to the navel points on the stomach and the spleen and gallbladder meridians (total 24 points)	Distillation (wild ginseng, Y) Alcohol immersion (BUM, Y)	n.r.	0.2 mL each (total 5 mL)	n.r.	n.r.	None

Kim et al., 2009 [11]	EAP, fixed	10 sessions (twice a wk for 5 wks)	ST25, CV4, CV6, and GB26	Distillation (Y)	n.r.	0.5 mL × 4 (total 2 mL)	n.r.	n.r.	Diet therapy + EA + HM + exercise

Pharmacopuncture classification according to the treatment rationale.

^*∗*^Pharmacopuncture method was classified into three categories based on the levels of individualization: “fixed” means all patients receive the same treatment at all sessions, “partially individualized” means using a fixed set of points to be combined with a set of points to be used flexibly, and “individualized” means each patient receives a unique and evolving diagnosis and treatment [38]. ^†^Pharmacopuncture point LI5 refers to 5th point of large intestine meridian and extra points have different nomenclature (e.g., Ex-UE3 means 3rd extra point in upper extremity). Ashi points mean local pain points; ^‡^guideline for extraction methods is based on the text book published in Korean Pharmacopuncture Institute [1]; ^§^1st: 3–7 days before 1st menstruation; 2nd: within 2 days after the start of 1st menstruation; 3rd: after the end of 1st menstruation; 4th: 3–7 days before 2nd menstruation; 5th: within 2 days after the start of 2nd menstruation.

A: acupuncture; BUM: *Calculus Bovis*.*Fel Ursi*.*Moschus*; CCP: *Coptis chinensis* pharmacopuncture; CS: Carthami-Semen; d: days; EA: electroacupuncture; EAP: *Ephedra sinica* Stapf-*Aconitum carmichaeli* Debx. pharmacopuncture; EST: electrical stimulation therapy; FES: functional electrical stimulation; G: gauge; HHT: Hwangryunhaedok-tang; HM: herb medicine; HP: hot pack; HPP: *Hominis Placenta* Pharmacopuncture; ICT: interferential current therapy; IR: infrared radiation; N: no; n.r.: not reported; PB: paraffin bath; PT: physical therapy; SSP: silver spike point; TENS: transcutaneous electrical nerve stimulation; wk: week; wks: weeks; WM: western medicine; Y: yes.

**Table 3:** Risk of bias (ROB) assessment^*∗*^.

Author, year	Random sequence generation	Allocation concealment	Blinding			Incomplete outcome data	Selective reporting
			Patient blinding	Practitioner blinding	Outcome assessor blinding		
Seo, 2013 [21]	Y	Y	U	N	Y	Y	Y
Lee, 2013 [19]	U	U	N	N	U	Y	Y
Lim, 2013 [9]	Y	U	U	U	U	Y	Y
Lee et al., 2012 (1) [22]	Y	U	N	N	U	Y	Y
Lee et al., 2012 (2) [23]	U	U	N	N	N	Y	Y
Park et al., 2011 (1) [12]	Y	Y	Y	Y	Y	Y	Y
Woo et al., 2011 [24]	Y	U	N	N	N	Y	Y
Kim et al., 2011 (1) [25]	U	U	N	N	N	N	Y
Jeong et al., 2011 [26]	U	U	N	N	N	N	Y
Jun et al., 2011 [27]	Y	Y	N	N	N	Y	Y
Im et al., 2011 [28]	U	U	N	N	U	N	Y
Park et al., 2011 (2) [29]	Y	U	N	N	N	Y	Y
Kim, 2011 (2) [10]	U	U	Y	U	U	N	Y
Kim et al., 2010 (1) [30]	U	Y	Y	Y	Y	Y	Y
Kim et al., 2010 (2) [37]	U	U	Y	U	U	N	Y
Noh et al., 2009 [18]	Y	U	Y	Y	U	Y	Y
Kim et al., 2009 [11]	Y	N	Y	N	U	N	Y
Song et al., 2009 [31]	U	U	N	N	U	Y	Y
Shin et al., 2009 [13]	Y	U	N	N	U	Y	Y
Choi et al., 2009 [14]	U	U	N	N	U	Y	Y
Kang et al., 2008 [32]	U	U	N	N	U	Y	Y
Park et al., 2008 [20]	Y	U	Y	N	Y	Y	Y
Kim et al., 2008 [36]	Y	U	Y	U	Y	Y	Y
Lee et al., 2007 [33]	U	U	N	N	U	Y	Y
Kim et al., 2006 [15]	U	U	Y	U	Y	Y	Y
Park et al., 2006 [34]	U	U	N	N	U	Y	Y
Lim et al., 2005 [16]	U	U	N	N	U	Y	N
Lee et al., 2005 [17]	U	U	Y	U	U	U	Y
Bae and Park, 2004 [35]	U	U	Y	Y	U	Y	Y

^*∗*^Based on the guidelines from the Cochrane Back Review Group [39]; “Y” indicates “yes (low risk of bias)”; “U,” “unclear”; “N,” “no (high risk of bias).”
